# Metabolic Reprogramming for Body Adaptation and Inflammatory Control in Eccentric Damaging Exercise: Comprehensive Molecular Insights From Repeated Downhill Running

**DOI:** 10.1002/mco2.70480

**Published:** 2025-11-14

**Authors:** Amir Mohammad Malvandi, Gianluca Vernillo, Veronica Sansoni, Martina Faraldi, Chiara Verdelli, Giuseppe Coratella, Giorgio Varesco, Vianney Rozand, Krystian Wochna, Laurent Mourot, Giovanni Lombardi

**Affiliations:** ^1^ Laboratory of Experimental Biochemistry & Advanced Diagnostics IRCCS Ospedale Galeazzi‐Sant'Ambrogio Milano Italy; ^2^ Department of Biomedical Sciences For Health Università Degli Studi di Milano Milano Italy; ^3^ Department of Medicine Université de Montreal Montreal Canada; ^4^ Université Marie et Louis Pasteur, SINERGIES Besançon France; ^5^ Chair of Sport Kinesiology Poznań University of Physical Education Poznań Poland

1

Dear Editor,

Exercise triggers metabolic adaptations that enhance cellular function and resilience, primarily through energy‐demanding processes. It stimulates key pathways like glycolysis, the tricarboxylic acid cycle (TCA), and oxidative phosphorylation, which are vital for muscle contraction and recovery. Studies on specific metabolites indicate that aerobic exercise enhances oxidative metabolism, whereas anaerobic and resistance exercises increase lactate production and stimulate muscle protein synthesis. Eccentric exercise, such as downhill running, leads to unique metabolic changes, marked by increased muscle damage markers such as creatine kinase (CK), myoglobin (MB), and lactate dehydrogenase (LDH); and inflammation (Cox‐2, iNOS). Although targeted studies have made progress, understanding the overall molecular dynamics of eccentric exercise remains limited [1]. The liquid chromatography with high‐resolution mass spectrometry (HRMS)‐based untargeted total small‐molecule profiling provides detailed insights into these changes [2], potentially guiding strategies to reduce muscle damage and enhance recovery [3].

This study investigated the biochemical and metabolic responses to repeated bouts of eccentric exercise, specifically downhill running, revealing distinct molecular trends between the first and second bouts (Figure 1A). The experimental protocol was effective in inducing muscle damage, as evidenced by a significant reduction in maximum voluntary isometric contraction (MVIC) 24 h post‐exercise (Figure 1A). These data were confirmed at the molecular level, as a notable increase in CK activity and MB concentrations was observed after exercise, with a significant reduction in the second bout (Figure 1B), indicative of reduced muscle damage—a hallmark of the repeated bout effect (RBE). LDH activity increased immediately postexercise in both bouts, with a faster recovery observed in the second bout, suggesting reduced cellular stress and faster recovery during the second bout (Figure 1B). Inflammatory markers showed minimal changes, with IL‐6 briefly detected postexercise and TNFα levels remaining stable (Figure 1B), indicating a controlled inflammatory response potentially influenced by prior adaptation [4].

**FIGURE 1:**
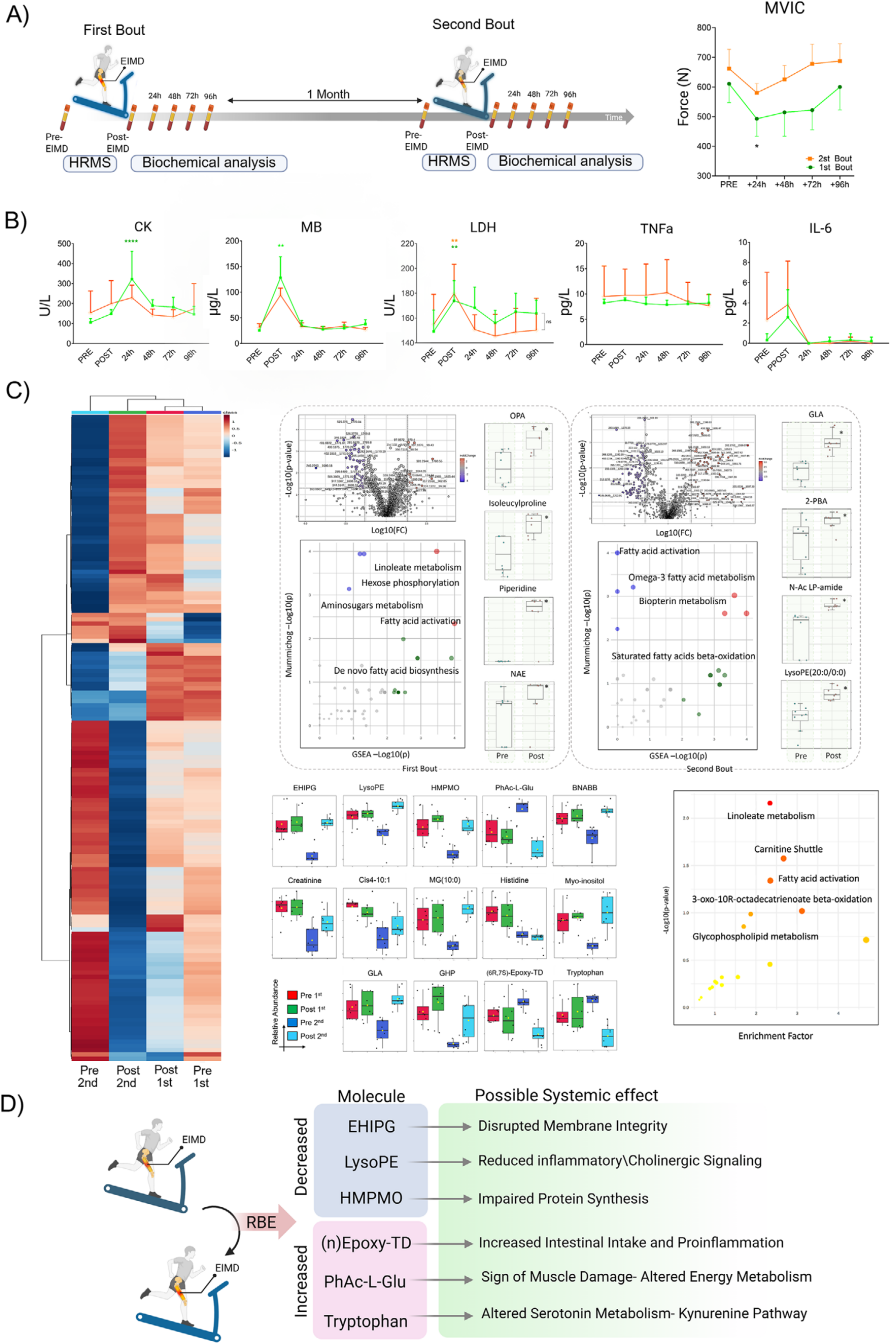
(A) Experimental design, sampling time‐points, and analysis. The exercise consisted of two downhill bouts of running (30 min; 10 km·h^−1^; −20%) interspersed by 4 weeks. On the right side of this part, the graph presents the maximum voluntary isometric contraction (MVIC) measurements before (pre) and at different time‐points after the bouts. Green represents the first bout, and orange represents the data for the second bout. (B) Serum biochemical evaluations of the participants. Data are presented in mean ± SD for the first bout in green squares, and the orange state for the second bout. (C) Global molecular profiling of serum of two consecutive eccentric damaging exercise bouts; at left of this panel, heatmap illustration of clustered (Euclidean‐Ward) identified molecular features in the group (first is first bout, second is second bout). Data are presented as the mean of all participants in each group. On the upper right, Vulcano plots of fold change (logarithmic) of molecular feature against *p*‐value (logarithmic) for the first and second bouts (post vs. pre); GSEA‐Mummichog integrated metabolic pathway enrichment analysis for the first bout. Levels of top hits identified metabolites for the bout. At the bottom of the central panel, top hits resulting from ANOVA are presented, demonstrating the metabolic dynamics of body adaptation to the repeated bout of eccentric damaging exercise. At the bottom right, the enrichment analysis, performed using the Mummichog algorithm, is presented. (D) Adaptive response to damaging eccentric exercise. This panel shows molecules that are observed to reduce or increase in participants after 1 month from the first bout of damaging eccentric exercise, returning to initial levels after the second bout of exercise. This adaptive response can indirectly help muscle regeneration and overall body resilience. (6R,7S)‐Epoxy‐TD, (6R,7S)‐epoxy‐tetradecadienoic acid; 2‐PBA, 2‐Phenylbutyric acid; BNABB, *N*‐benzoyl‐*N*‐(4‐aminobutyl)benzamide; Cis4‐10:1 MG(10:0), Cis‐4‐decenoic acid monoglyceride (10:0); CK, creatine kinase; EHIPG, ethyl 2‐hydroxy‐3‐(3‐indolyl)propanoate carboxylate; GHP, glutathione hydroperoxide; GLA, gamma‐linolenic acid; HMPMO, *N*‐(3‐hydroxyphenyl)malonamic acid; HRMS, high‐resolution mass spectrometry; IL‐6, interleukin‐6; LDH, lactate dehydrogenase; LysoPE (20:0/0:0), lysophosphatidylethanolamine with a 20:0 fatty acid chain and no second fatty acid chain; LysoPE, lysophosphatidylethanolamine; MB, myoglobin; N‐AC‐LP amid, *N*‐acetyl‐l‐cysteine‐lipid amid; NAE, *N*‐acylethanolamine; OPA, (Z)‐2‐octylpent‐2‐enedioic acid; PhAc‐L‐Glu, phenylacetic acid‐l‐glutamate; TNFα, tumor necrosis factor‐α. Created in BioRender. Lombardi (2025). https://BioRender.com/b2hkbpg.

High‐resolution small‐molecular profiling revealed profound metabolic shifts associated with exercise‐induced fatigue and recovery (Figure 1C). In this study, we focused on comparing *immediate pre‐* and postexercise samples across both bouts, which demonstrated distinct metabolic responses, underscoring the adaptive nature of repeated eccentric stress. The volcano plot analysis identified significant metabolites that underwent substantial changes postexercise (Figure 1C), with pathway enrichment analysis revealing key metabolic shifts. The first bout was characterized by alterations in linoleate metabolism, hexose phosphorylation, fatty acid (FA) activation, aminosugar metabolism, and de novo FA biosynthesis (Figure 1C). These pathways reflect an acute metabolic response involving energy utilization, lipid metabolism, and structural remodeling to counteract exercise‐induced damage.

The second bout continued to exhibit some of these metabolic perturbations. Still, it introduced new adaptive pathways compared with the first bout, including Omega‐3 FA metabolism, protein metabolism, and saturated FAs β‐oxidation (Figure 1C), revealing metabolic rewiring states in the body. The emergence of Omega‐3 FA metabolism highlights an increased reliance on anti‐inflammatory and membrane‐stabilizing mechanisms, consistent with a protective adaptation that mitigates muscle damage. The activation of protein metabolism observed in serum may indirectly reflect enhanced muscle remodeling and turnover (Figure 1C); however, this interpretation should be made with caution, as serum‐based measurements do not directly represent changes occurring within skeletal muscle tissue [5]. The upregulation of saturated FAs β‐oxidation further underscores a shift toward lipid‐based energy metabolism, a characteristic adaptation in repeated exercise bouts to optimize endurance and recovery efficiency [6].

A detailed comparison of serum molecular profiles before and after both exercise sessions revealed a distinctive pattern of molecular dynamics resulting from the RBE. Enrichment analysis of metabolic pathways indicated linoleate metabolism as the most significantly modified, followed by the carnitine shuttle, glycerophospholipid metabolism, 3‐oxo‐10R‐octadecatrienoate β‐oxidation, and FA activation. These findings highlight the progressive reprogramming at the systemic level of metabolic networks, favoring lipid metabolism and energy efficiency upon repeated exposure to eccentric exercise stress.

Examining the participants’ serum molecular dynamics provides deeper insights into these adaptations. The persistence of alterations in pathways such as 3‐oxo‐10R‐octadecatrienoate β‐oxidation and FA activation suggests a sustained demand for lipid metabolism, possibly reflecting a long‐term adaptation to eccentric exercise. The detection of ethyl‐2‐hydroxy‐3‐(3‐indolyl) propanoate glucoside, a derivative of tryptophan metabolism, underscores the interplay between muscle function and central nervous system activity (Figure 1D). Tryptophan metabolism can influence serotonin synthesis, thereby linking metabolic adaptations to the regulation of neuromuscular fatigue and psychological stress resilience.

Additionally, the increased levels of LysoPE (0:0/20:0) immediately after exercise in serum may indirectly indicate the role of lipid metabolism in preserving muscle membrane integrity and facilitating post‐exercise recovery. LysoPEs contribute to membrane remodeling and generate signaling molecules essential for managing inflammation and muscle repair (Figure 1D). The upregulation of *N*‐[(4‐hydroxy‐3‐methoxyphenyl)methyl]octanamide, associated with the phenylpropanoid pathway, can suggest an interaction between muscle activity and gut health, reinforcing the concept of a gut–muscle axis influencing recovery and metabolic balance.

Further, our systemic molecular profiling revealed the possible presence of exogenous metabolites in participants’ serum, such as 2‐benzamido‐*N*‐(1‐[(naphthalen‐1‐yl)amino]‐1‐oxobutan‐2‐yl)benzamide, *N*‐(3‐acetamidopropyl)pyrrolidin‐2‐one, α‐ or β‐triticene, and piperidine, indicating potential alterations in gut permeability induced by exercise. These changes highlight the comprehensive impact of eccentric exercise on the entire body, as metabolic shifts extend beyond muscle tissue to encompass broader physiological systems. The elevation of *N*(2)‐phenylacetyl‐l‐glutaminate reflects active detoxification pathways, highlighting the body's effort to clear metabolic waste products generated during physical exertion [7].

These findings suggest that repeated eccentric exercise promotes a reorganization of metabolic pathways, favoring lipid metabolism, efficient energy utilization, and enhanced recovery processes at the systemic body level. The identified metabolites and pathways provide valuable serum biomarkers for monitoring exercise adaptation and recovery. The shift toward Omega‐3 FA metabolism and the persistence of lipid‐based energy utilization can potentially be targeted by nutritional interventions, such as Omega‐3 supplementation, that could further enhance these adaptations. Moreover, the observed gut‐derived metabolites highlight the importance of considering gut health in designing personalized recovery strategies [8].

Considering key and significantly observed molecules from our analysis, we can hypothesize and suggest that enzymes linked to observed exercise‐induced metabolic perturbations are potential new serum biomarkers for assessing physiological stress and tissue damage from intense physical activity. Tryptophan hydroxylase (TPH), indoleamine 2,3‐dioxygenase (IDO), and tryptophan 2,3‐dioxygenase (TDO), associated with ethyl 2‐hydroxy‐3‐(3‐indolyl)propanoate glucoside and tryptophan, may reflect disruptions in serotonin and kynurenine pathways during prolonged exercise, which are linked to fatigue, inflammation, and oxidative stress. Lysophospholipase (LYPLA) and phospholipase A2 (PLA2), tied to LysoPE (0:0/20:0), could indicate membrane phospholipid breakdown in muscle or erythrocytes under mechanical or oxidative strain. The detoxification enzymes catechol‐O‐methyltransferase COMT and uridine diphosphate‐glucuronosyltransferases (UGT), involved in metabolizing *N*‐[(4‐Hydroxy‐3‐methoxyphenyl)methyl]octanamide and 4′,7‐Di‐O‐methylcatechin, may highlight catecholamine clearance and antioxidant responses critical to recovery. Glutamine synthetase (GS) and glutaminase (GLS), linked to *N*(2)‐phenylacetyl‐l‐glutaminate and glutamylhydroxyproline, could signal shifts in nitrogen balance and collagen turnover during muscle repair or catabolism. Enzymes like Acyl‐CoA dehydrogenase (ACAD) and enoyl‐CoA hydratases (ECH), acting on cis‐4‐Decenoic acid, might reflect mitochondrial dysfunction in FA oxidation during endurance overload, while FA desaturase 2 (FADS2)/elongase (gamma‐linoleic acid) could denote altered anti‐inflammatory lipid mediator synthesis. Histidine decarboxylase (HDC) and histidase, which are linked to histidine, may contribute to histamine‐driven inflammation or impaired carnosine synthesis in muscle fatigue. Lastly, monoglyceride lipase (MGLL), linked to MG (18:0/0:0/0:0), may indicate dysregulated lipolysis during energy depletion. These enzymes provide a multifaceted serum biomarker panel to assess metabolic strain, tissue damage, and recovery needs in response to damaging exercise, facilitating the optimization of training loads or the mitigation of injury risks.

In conclusion, this study presents a high‐resolution systemic molecular landscape of metabolic adaptation to repeated eccentric exercise. The findings reinforce the role of lipid metabolism in facilitating recovery, reducing muscle damage, and improving exercise efficiency. Understanding these molecular adaptations provides a foundation for optimizing exercise strategies, recovery interventions, and the development of potential biomarkers for monitoring of body physiological status. Our findings reflect systemic circulating signatures, their temporal proximity to muscle contraction, and the dominant role of muscle in exercise metabolism, supporting a biologically meaningful link to muscular responses. However, since our conclusions are limited by serum molecular profiling at a relatively small cohort, further research incorporating direct muscle tissue analysis and larger cohorts is needed to expand on these insights and establish broader applications of these findings.

## Author Contributions

A.M.M. designed the project, performed the experiments, analyzed the data, and wrote the manuscript. G.V. designed the project, performed the experiments, and edited the manuscript. V.S., M.F., and C.V. performed biochemical experiments. G.C., G.V., V.R., K.W., and L.M. performed the exercise experiments. G.L. designed the project, edited the manuscript, and supervised the project. All authors have read and approved the final manuscript.

## Funding

This research was supported by the Ultra‐Endurance Sports Science & Medicine Grant Program. This work was supported by the Italian Ministry of Health “Ricerca Corrente.”

## Ethics Statement

The institutional ethical committee approved the study (RCB number ID‐RCB: 2019‐A03012‐55). All participants were informed and provided consent to participate in this study. The protocol was designed in compliance with the Helsinki Declaration.

## Conflicts of Interest

The authors declare no conflicts of interest.

## Supporting information




Supplement information file 1 "mco270480‐sup‐0001‐SuppMat.docx"

## Data Availability

The Supporting Information for this work, which is available on the journal's website, provides a detailed description of our study design and the Materials and Methods used. The raw data of HRMS analysis are available online at https://doi.org/10.5281/zenodo.14204023.
