# Supplementary material for: Metabolic Reprogramming for Body Adaptation and Inflammatory Control in Eccentric Damaging Exercise: Comprehensive Molecular Insights From Repeated Downhill Running
Source: MedComm (2020). 2025 Nov 14;6(11):e70480. doi: 10.1002/mco2.70480 (PMC12617245; doi:10.1002/mco2.70480)
Supplement: Supplementary file 1 — Supplement information file 1 "mco270480‐sup‐0001‐SuppMat.docx" [file MCO2-6-e70480-s001.docx]

# Supplementary Materials

**Metabolic Reprogramming for Body Adaptation and Inflammatory Control in Eccentric Damaging Exercise: Comprehensive Molecular Insights from Repeated Downhill Running**

Amir Mohammad Malvandi^1^*, Gianluca Vernillo^2^, Veronica Sansoni^1^, Martina Faraldi^1^, Chiara Verdelli^1^, Giuseppe Coratella^2^, Giorgio Varesco^3^, Vianney Rozand^4^, Krystian Wochna^5^, Laurent Mourot^4^, Giovanni Lombardi^1,5^

^1^ Laboratory of Experimental Biochemistry & Advanced Diagnostics, IRCCS Ospedale Galeazzi-Sant'Ambrogio, Milano, Italy

^2^ Department of Biomedical Sciences for Health, Università degli Studi di Milano, Milano, Italy

^3^ Department of Medicine, Université de Montreal, Montreal, QC, Canada

^4^ Université de Franche-Comté, Sinergies, Besançon, France

^5^ Chair of Sport Kinesiology, Poznań University of Physical Education, Poznań, Poland

Author for correspondence:

**Amir M Malvandi PhD**

Laboratory of Experimental Biochemistry & Advanced Diagnostics

IRCCS Ospedale Galeazzi-Sant'Ambrogio

Via Cristina Belgioioso 173 - 20157, Milano, Italy

*Email: Amirmohammad.Malvandi@grupposandonato.it*

ORCID: 0000-0003-1243-2372

# Methods Cohort

Eighteen healthy male volunteers participated in this study (age, mean = 21.78 ± 2.15 years; BMI, mean = 22.90 ± 2.12 kg/m^2^), as previously reported [1]. For the present study, four of those volunteers returned to repeat the experimental protocol and were included in the analysis. The nine participants analyzed in the previous publication were distinct from the four participants included here. The included participants were recreational runners with less than four days of running cycles per week. The exclusion criteria were set at smoking, medication, and the presence of apparent musculoskeletal, neurologic, metabolic, or cardiovascular diseases. Moreover, participants were excluded if they were already familiarized with other types of eccentric exercises (e.g., strength training) and/or had regular physical activity involving substantial eccentric contractions within six months before the start date of this study. The consumption of anti-inflammatory drugs, aspirin and ibuprofen, caffeine, and alcohol within 48 h before the test was forbidden. Participants were instructed to keep a uniform type of diet and to avoid any strenuous exercise before and during the study.

# Experimental design

Each participant attended the laboratory on separate occasions. Room temperature and humidity were similar across sessions (21 °C, 45% RH). Participants first attended a familiarization session and then, one week later, the first experimental session. Participants who volunteered for the repeated experimental round came back 1-month after the first experimental session for the second and last experimental session. During the familiarization, participants performed a single 5-min downhill run, replicating the experimental settings on the same treadmill used for the experimental session. During the experimental session(s), participants performed a warm-up consisting of 10 min of light pedaling on a cycle ergometer (Monark 818E, Stockholm, Sweden). After 5 min, participants performed a 30-min downhill run at a running speed of 10 km·h-1 and a slope of -20% on a motorized treadmill (Medic 2855, Genin Medical, La Roque-d’Anthéron, France). The protocol was designed to induce muscle damage as previously described [1-3]. Blood sampling was performed before (PRE) and immediately after (POST) the downhill run, as well as 24h, 48h, 72h, and 96h after (Figure 1). Blood samples were collected using standard venipuncture from the antecubital vein in SST II Advance Vacutainer® and K2EDTA tubes (Becton, Dickinson & Co., Franklin Lakes, NJ, USA). The tubes were then centrifuged at 3000 × *g* for 15 min, at 4 °C, and the resulting serum and plasma were divided into smaller portions and stored at −80 °C until the analysis. The samples were thawed only once.

**Measurement of Maximum Voluntary Isometric Contraction (MVIC):**

MVIC of the right knee extensors was assessed with participants seated at 90° hip and knee flexion using a customized dynamometer (Legcontrol, Mtraining, France). The leg was secured 3 cm above the medial malleolus, and a waist belt minimized upper body movement. After a standardized warm-up (20 × 2-s contractions), participants performed two 4-s MVICs separated by 5 min rest. If the difference between trials exceeded 5%, additional attempts were made until two consecutive values differed by <5%. Force signals were recorded at 2 kHz (PowerLab 16/30, AD Instruments), and the highest value was used for analysis.

# Serum biochemistry evaluations

CK activity (CK_L assay) is determined by the reaction of creatine phosphate with adenosine diphosphate (ADP) to produce ATP. This is coupled to the hexokinase-G6PD (glucose-6- phosphate dehydrogenase) reaction, generating NADPH (reduced nicotinamide adenine dinucleotide phosphate). The concentration of NADPH is measured by the increase in absorbance at λ=340/596 nm. The measuring range is 15-1300 U/L. The assay is designed to have a limit of blank (LoB) of ≤ 10 U/L, a limit of detection (LoD) of ≤ 15 U/L, and a limit of quantitation (LoQ) of ≤ 25 U/L. The maximum coefficient of variation (CV) is 2.4%.

LDH activity (LDLP assay) measurement is based on the LDH-mediated conversion of L-lactate to pyruvate in the presence of nicotinamide adenine dinucleotide (NAD). The enzymatic activity of LD is proportional to the rate of production of NADH (reduced NAD), which is determined by measuring the increase in absorbance at λ=340/410 nm. The measuring range is 14-750 U/L. The assay is designed to have a LoB < LoD, LoD ≤ 20 U/L, and LoQ≤ 25 U/L. The maximum CV is 1.0%.

CK and LDH activity were tested in serum samples using an Atellica® CH Analyzer manufactured by Siemens Healthineers Corp. in Erlangen, Germany. The instrument was calibrated and quality controls were performed according to the manufacturer’s instructions.

Myoglobin was tested in serum samples using an an Atellica® NEPH 630 nephelometer (Siemens Healthineers, Germany) with a N Latex Myoglobin kit (Siemens Healthineers). Polystyrene beads coated with specific myoglobin antibodies bind to myoglobin in serum samples. The resulting immune complexes scatter light, and the intensity of this scattered light is proportional to the concentration of myoglobin in the sample. Nephelometer was used to determining specific protein concentration in fluids by measurement of the scattered light intensity in a fixed angle of 13–24° with a wavelength of 840 ± 10 nm. The assay is designed to have LoD < 6.24 µg/L and a CV <10%. Quality control was performed following manufacturer’s instruction prior to samples measurment.

# Multiplex Luminex-based cytokine measurement

In the study, we used a 5-plex Luminex performance panel (FCSTM18-05, Biotechne Corp., Minneapolis, MN, US) to analyze inflammatory cytokines in the subjects’ sera. The sensitivities for interferon (IFN)α, IFNβ, interleukin (IL)-1β, IL-6, and tumor necrosis factor (TNF)-α were 0.29 pg/mL, 0.31 pg/mL, 0.25 pg/mL, 0.38 pg/mL, and 0.62 pg/mL, respectively. Samples were run in duplicate on a Luminex® MagPix®, and results were calculated using Bioplex™ Manager software v.6, with a recovery range of 80%-120%.

# Metabolite extraction procedure

Total small molecules were isolated from 50 µL of serum samples using the Methyl tert-butyl ether (MTBE) based biphasic extraction protocol. Each collected sample’s serum was treated with proteinase K (1 U/1 mg of protein) for 15 min at 50°C, as per previously described protocols [4, 5], to enhance the release of metabolites from the protein matrix. Next, biphasic extraction was performed on the samples using MTBE: MeOH: H2O (4:4:0.5 v:v). The samples were then stored overnight at -20°C, sonicated for 20 min, and centrifuged to remove the protein phase. Subsequently, the samples were dried under N2 gas at RT, resuspended in ACN:MeOH:H2O (2:2:1 v:v), and analyzed using high-resolution liquid chromatography-mass spectrometry (HRMS). All chemicals used were LC-MS grade and purchased from Merck Italy unless otherwise specified.

# Untargeted high-resolution metabolomics analysis

The metabolites extracted were analyzed using a high-resolution mass spectrometer (Orbitrap Velos Pro, Thermo Fisher Inc., Waltham, MA, US) connected to a high-performance liquid chromatography system (Surveyor, Thermo Fisher). Each sample was analyzed at least twice using both positive and negative scan data-dependent analysis (DDA) modes (MS1 at 30000 FWHM, MS2 linear trap Velos Pro). The chromatography gradient was set for 30 minutes, beginning at 2% and reaching 90% methanol (1% Formic acid) on a Kinetex® 2.6 µm polar C18 100 Å (Phenomenex, Inc., Torrance, CA, US) HPLC column. Isocratic washing for 2 min was performed between each run, and blank runs were carried out after each sample batch analysis. Throughout the analysis, samples were maintained at +8°C and the column was kept at 45°C. The ion source used was a standard electrospray (ESI) from Thermo Fisher, and the liquid flow rate was set to 200 µL/min.

# AI-based quality control (AI-QC)

We evaluated the accuracy of the HRMS analysis using the traditional internal control recovery rate and an AI-based quality control platform developed by ISB srl (Milano, Italy), as previously described [4]. For this study, we set the accepted vectorial correlation rate at 800. This approach provided greater reproducibility and reduced variability across different samples and analyses. The algorithm evaluates external control and all features simultaneously, comparing them with quality control and blank samples.

# Data analysis and interpretation

All data features were obtained from aligned analysis using the XCMS package (v. 3) within the R software platform (v. 4.4.0) for initial screening and statistical differences analysis. The primary analysis was conducted at MS1 level, involving principal component analysis (PCA) and T-student tests on inter-quantile normalized data expressed in a natural logarithmic scale. The most significant features were then verified at the MS2 level using a locally constructed library on the NIST software platform, and these confirmed MS2 features were utilized for pathway enrichment analysis.

Next, spectral analysis and annotation at MS1 and MS2 levels were performed using the MetaboAnalyst Pro (v.2) platform. The hit molecules at MS1 annotated with HMDB and KEGG (released date 01/14/2024) were then confirmed with the MS2 alignments according to the predisposed function of the platform [6]. Since the experimental design consisted of two groups of pre- and post-exercise for the first and second bouts of exercise sections, the analysis was performed either between the pre- and post-exercise of each single bout or all groups together. Volcano plots of fold change and *p*-value were used to compare two groups and ANOVA was used to reveal the most differentially expressed small molecules.

In all analyses, the features common with blank and internal controls (QC), and the features directly connected with food consumption without biological process, were filtered out from all samples; therefore, the blank samples were excluded from the comparisons. All statistical analyses were performed on interquartile normalized and log transformed data in robust conditions, and filtering was applied to increase the reliability of the data; the threshold of relative standard deviation (RSD = SD/mean) was set at 20%, variance filter was 5% of Interquartile range, and abundance filter was applied at 5% of mean intensity value [7].

# References

1. Varesco G, Coratella G, Rozand V, Cuinet B, Lombardi G, Mourot L, Vernillo G: **Downhill running affects the late but not the early phase of the rate of force development**. *Eur J Appl Physiol* 2022, **122**(9):2049-2059.

2. Coratella G, Varesco G, Rozand V, Cuinet B, Sansoni V, Lombardi G, Vernillo G, Mourot L: **Downhill running increases markers of muscle damage and impairs the maximal voluntary force production as well as the late phase of the rate of voluntary force development**. *Eur J Appl Physiol* 2024, **124**(6):1875-1883.

3. Khassetarash A, Vernillo G, Kruger RL, Edwards WB, Millet GY: **Neuromuscular, biomechanical, and energetic adjustments following repeated bouts of downhill running**. *J Sport Health Sci* 2022, **11**(3):319-329.

4. Malvandi AM, Halilaj E, Faraldi M, Mangiavini L, Cristoni S, Leoni V, Lombardi G: **Enhanced molecular release from elderly bone samples using collagenase I: insights into fatty acid metabolism alterations**. *J Transl Med* 2024, **22**(1):143.

5. Wawrzyniak R, Kosnowska A, Macioszek S, Bartoszewski R, Jan Markuszewski M: **New plasma preparation approach to enrich metabolome coverage in untargeted metabolomics: plasma protein bound hydrophobic metabolite release with proteinase K**. *Sci Rep* 2018, **8**(1):9541.

6. Pang Z, Lu Y, Zhou G, Hui F, Xu L, Viau C, Spigelman AF, MacDonald PE, Wishart DS, Li S *et al*: **MetaboAnalyst 6.0: towards a unified platform for metabolomics data processing, analysis and interpretation**. *Nucleic Acids Res* 2024, **52**(W1):W398-W406.

7. Hackstadt AJ, Hess AM: **Filtering for increased power for microarray data analysis**. *BMC Bioinformatics* 2009, **10**:11.
